# Associations of *ABHD2* Genetic Variations with Risks for Chronic Obstructive Pulmonary Disease in a Chinese Han Population

**DOI:** 10.1371/journal.pone.0123929

**Published:** 2015-04-16

**Authors:** Li Liu, Xiangshun Li, Rui Yuan, Honghong Zhang, Lixia Qiang, Jingling Shen, Shoude Jin

**Affiliations:** 1 Department of Histology and Embryology, Harbin Medical University, Harbin, Heilongjiang Province, 150018, China; 2 Division of Respiratory Disease, The Fourth Hospital of Harbin Medical University, Harbin, Harbin, Heilongjiang Province, 150001, China; Zhongshan Hospital Fudan University, CHINA

## Abstract

The human α/β hydrolase domain-containing protein 2 gene (*ABHD2*) plays a critical role in pulmonary emphysema, a major subset of the clinical entity known as chronic obstructive pulmonary disease (COPD). Here, we evaluated genetic variation in the *ABHD2* gene in a Chinese Han population of 286 COPD patients and 326 control subjects. The rs12442260 CT/CC genotype was associated with COPD (*P* < 0.001) under a dominant model. In the former-smoker group, the rs12442260 TT genotype was associated with a decreased risk of developing COPD after adjusting for age, gender and pack-years (*P* = 0.012). Rs12442260 was also associated with pre-FEV1 (the predicted bronchodilator forced expiratory volume in the first second) in controls (*P* = 0.027), but with FEV1/ forced vital capacity (FVC) ratios only in COPD patients (*P* = 0.012) under a dominant model. Results from the current study suggest that *ABHD2* gene polymorphisms contribute to COPD susceptibility in the Chinese Han population.

## Introduction

Chronic obstructive pulmonary disease (COPD), including chronic pulmonary emphysema and chronic bronchitis, is one of the leading causes of morbidity and mortality worldwide. It is characterized by a partially reversible airflow limitation, which is usually progressive and associated with abnormal inflammatory responses of the lungs when exposed to noxious particles or gases [1–4]. According to the World Health Organization, by 2020, COPD may become the fourth most common single cause of death and is expected to be the third-leading cause of death in developed countries [5–8]. The global incidence of COPD is approximately 9〜10% in adults aged ≥40 years of age [9], and 8.2% in this age group in China [10]. Accounting for 80–90% of COPD cases, cigarette smoking is by far the major risk factor for COPD. However, only 10〜15% of smokers develop clinically significant COPD, and some former smokers and non-smokers can develop COPD [11–14]. Current evidence suggests that inherited risks influence COPD susceptibility, as observed with various cancers and other diseases types. Results from family studies, case-control studies, and recent hypothesis-free genome-wide association (GWA) studies have highlighted the role of genetic factors in COPD development [15, 16], although the molecular genetics of underlying COPD pathogenesis are not completely understood. At present, many studies suggest that COPD, a complex polygenic disease, is caused by interaction between genetic and environmental factors. Thus, the identification of genetic and environmental risk factors that cause an increased risk of COPD may be helpful in improving its treatment.

The α/β hydrolase domain-containing protein 2 gene (*ABHD2*, MIM612196) is located at 15q26.1 and is a member of the α/β hydrolase superfamily that was identified in a genetic screen of human emphysematous tissue [17]. *ABHD2* encodes a protein containing an alpha/beta hydrolase fold, which serves a catalytic domain in a wide range of enzymes. Thus, human *ABHD2* should have multiple functions in maintaining the structural integrity of lungs, as observed with mouse *Abhd2* [18].

Results from previous study revealed that the mouse *Abhd2* is expressed in vascular smooth muscle cells (SMCs) and that enhanced migration occurs with cultured vascular SMCs from *Abhd2*-deficient mice [19]. Enhanced neointimal hyperplasia was observed in *Abhd2*-deficient mice, using an experimental vascular cuff placement injury model [20]. Previously, we used Abhd2-deficient mice obtained by gene trap mutagenesis to examine the role(s) of Abhd2 in mouse lungs. Our results showed that derangement of alveolar phospholipid metabolism could induce emphysema and that Abhd2 played a critical role in maintaining lung structural integrity [18]. Recently, Tsuyoshi Yoshida *et al*. [21] reported an association between the human *ABHD2* gene and colorectal cancer, although the molecular function of *ABHD2* in driving colorectal cancer remains uncertain. Maryam Shahdoust *et al*. [22] recruited 13 normal smokers and 9 non-smokers to identify differentially expressed genes between 2 groups and assessed the effects of cigarette smoking on large airway epithelia cells. Their results showed that *ABHD2* was more highly expressed in smokers, compared with a control group consisting of non-smokers.

The aim of the present study was to investigate whether single-nucleotide polymorphisms (SNPs) in the *ABHD2* gene are related to COPD in the Chinese Han population using a large panel of samples obtained from the patients with COPD and control subjects.

## Results

A total of 286 patients with COPD and 326 normal controls were enrolled in this study. The characteristics of the participants enrolled in this study are summarized in Table 1. The mean age was 67.60 years (±9.59 years) for patients with COPD and 66.40 years (±9.76) for control subjects. Males comprised 65.7% of the COPD group, compared with 64.1% in the control groups. Thus, there were no significant differences in the age distribution (*P* = 0.137) or gender (*P* = 0.675) between the COPD and control groups. Patients with COPD had greater average smoking exposure (32.80 vs. 25.57 pack-years, *P* < 0.001), and significantly worsened lung functions than control subjects, in term of the predicted FEV1 percent (69.32% vs. 104.61%, *P* < 0.001) and FEV1/FVC ratio (0.58 vs. 0.84, *P* < 0.001).

**Table 1:** Characteristics of COPD patients and healthy controls.

Variable	Case subjects(n = 286)	Control subjects(n = 326)	*P* Value
Mean age (years)^a^	67.60±9.59	66.40±9.76	0.137^b^
Male % (n)	65.7 (188)	64.1(209)	0.675^c^
Current smoking status			
Non-smoker % (n)	35.0 (100)	49.1(160)	
Former smoker % (n)	30.4 (87)	24.5 (80)	0.002^c^
Current smoker % (n)	34.6 (99)	26.4 (86)	
Pack-years^a^	32.80±10.77	25.57±8.90	<0.001^b^
FEV1 percentage of predicted^a^, %	69.32±9.14	104.61±7.07	<0.001^b^
FEV1/FVC^a^, L	0.58±0.07	0.84±0.04	<0.001^b^

ND = not done; FEV1, forced expiratory volume in 1 second; FVC, forced vital capacity.

^a^Mean (±SD).

^b^ *P* value was calculated using the *t* test.

^c^ *P* value was calculated using the χ^2^ test.

The relative frequencies of *ABHD2* SNP polymorphisms observed in patients enrolled in this study are shown in Table 2. The overall call rates for each SNP exceeded 97%. The distribution of genotypes in the control group was consistent with the Hardy-Weinberg equilibrium (HWE; *P* > 0.05). No significant differences SNP allele frequencies were observed between patients with COPD and control subjects, except for rs12442260 (*P* = 0.001). In addition, the genotype frequencies between the COPD and controls groups for 6 SNPs were analyzed. A statistically significant association was found only between the rs12442260 polymorphism and the risk for developing COPD. Compared to TT homozygotes at the rs12442260 locus, CT heterozygotes showed a significantly increased risk for COPD (OR = 1.84, 95% CI = 1.31–2.59; *P* < 0.001, *P*_adjust_ < 0.001), as did CC homozygotes (OR = 1.78, 95% CI = 1.04–3.06; *P* = 0.035, *P*_adjust_ = 0.025). The rs12442260 genotype exhibited significance under a dominant genetic model (CC + CT vs. TT: OR = 1.83, 95% CI = 1.32–2.53; *P* < 0.001), indicating that carriers of the C allele had an 83% increased risk for developing COPD compared with homozygotes. However, such an elevated risk was not observed, under a recessive genetic model (CC vs. CT + TT: OR = 1.32, 95% CI = 0.79–2.21; *P* = 0.282). In addition, individuals with the rs293377 CG genotype had a 43% increased risk for developing COPD compared with homozygotes, as determined by comparative analyses conducted with or without adjust for age, gender, and current smoking status and all patients in the COPD and control groups (GC vs. CC: *P*_adjust_ = 0.018). The frequency of the GC allele was increased in patients with COPD by 61.5%, compared to control subjects. No significant differences were observed between the COPD and control groups with respect to the other SNPs.

**Table 2:** Risk associated with the six polymorphisms.

SNP	Genotype / Allele	Case n = 286 (%)	Control n = 326 (%)	Call rate (%)	*P*-HWE	OR^b^ (95% CI)	*P* value	*P* ^a^
rs293379	T	205 (36.6)	254 (39.2)	98.7	0.265		0.355	
	C	355 (63.4)	394 (60.8)					
	TT	31 (11.1)	45 (13.9)			0.75 (0.44–1.26)	0.279	0.273
	CT	143 (51.1)	164 (50.6)			0.95 (0.67–1.34)	0.619	0.854
	CC	106 (37.8)	115 (35.5)			1		
rs293377	G	272 (48.1)	298(46.6)	98.5	0.152		0.604	
	C	294 (51.9)	342 (51.9)					
	GG	49 (17.3)	63 (19.7)			1.10 (0.67–1.81)	0.703	0.857
	GC	174 (61.5)	172 (53.7)			1.43 (0.97–2.12)	0.071	0.018
	CC	60 (21.2)	85 (26.6)			1		
rs16942690	G	172 (31.0)	211 (33.2)	97.2	0.800		0.433	
	A	382 (69.0)	425 (66.8)					
	GG	23 (8.3)	36 (11.3)			0.71 (0.40–1.27)	0.249	0.230
	GA	126 (45.5)	139 (43.7)			0.91 (0.71–1.61)	0.942	0.801
	AA	128 (46.2)	143 (45.0)			1		
rs293381	T	225 (39.5)	267 (41.5)	99.2	0.055		0.482	
	C	345 (60.5)	377 (58.5)					
	TT	28 (9.8)	47 (14.6)			0.69 (0.40–1.19)	0.184	0.184
	CT	169 (59.3)	173 (53.7)			1.13 (0.79–1.62)	0.493	0.505
	CC	88 (30.9)	102 (31.7)			1		0.182
rs12442260	C	210 (37.0)	182 (28.2)	99.2	0.140		0.001	
	T	358 (63.0)	464 (71.8)					
	CC	35 (12.3)	31 (9.6)			1.78 (1.04–3.06)	0.035	0.025
	CT	140 (49.3)	120 (37.2)			1.84 (1.31–2.59)	<0.001	<0.001
	TT	109 (38.4)	172 (53.2)			1		
rs729707	G	231 (41.4)	289 (44.5)	98.7	0.104		0.284	
	A	327 (58.6)	361 (55.5)					
	GG	31 (11.1)	57 (17.5)			0.64 (0.38–1.09)	0.098	0.144
	AG	169 (60.6)	175 (53.9)			1.14 (0.79–1.64)	0.493	0.513
	AA	79 (28.3)	93 (28.6)			1		

SNP, single nucleotide polymorphism; HWE, Hardy-Weinberg equilibrium; OR, odds ratio; CI, confidence interval.

^a^ Adjusted for age, gender, and smoking status.

^b^ Odds ratios are relative to the major homozygous genotype.

Correlations between COPD and smoking status are well documented. A smoking status stratification analysis was performed to eliminate potential confounding effects caused by differences in smoking history. As shown in Table 3, we found that none of the SNPs studied were significantly associated with COPD in non-smokers (n = 260) or current smokers (n = 185). However, SNP analysis with samples from former smokers (n = 167) showed that the rs12442260 polymorphism was significantly associated with COPD after adjusting for age, gender and pack-years (CT vs. TT: OR_adjust_ = 0.401, 95% CI_adjust_ = 0.20–0.82; *P*_adjust_ = 0.012), and the TT genotype was associated with a decreased risk for COPD. Under the assumption of a dominant model of inheritance, there was an association between the rs12442260 polymorphism and COPD (CC + CT vs. TT: OR = 2.07, 95% CI = 1.10–3.89; *P* = 0.022).

**Table 3:** Association between SNPs in the *ABHD2* and COPD by smoking status-stratified analysis.

Reference	Genotypes	Non-smokers (n = 260)			Former smokers (n = 167)			Current smokers (n = 185)		
SNP ID		COPD n = 100(%)	Control n = 160(%)	*P* ^a^	COPD n = 87(%)	Control n = 80(%)	*P* ^b^	COPD n = 99(%)	Control n = 86(%)	*P* ^b^
rs293379	TT	10 (10.0)	19 (11.8)	0.504	9 (11.1)	12 (15.4)	0.608	12 (12.1)	14 (16.3)	0.530
	CT	51 (51.0)	87 (54.4)	0.390	39 (48.2)	38 (48.7)	0.820	53 (53.5)	39 (45.3)	0.306
	CC	39 (39.0)	54 (33.4)		33 (40.7)	28 (35.9)		34 (34.3)	33 (38.4)	
rs293377	GG	14 (14.0)	28 (17.5)	0.954	16 (19.1)	18 (24.3)	0.938	19 (19.2)	17 (19.8)	0.666
	CG	70 (70.0)	98 (61.3)	0.196	48 (57.1)	35 (47.3)	0.157	56 (56.6)	39 (45.3)	0.150
	CC	16 (16.0)	34 (21.2)		20 (23.8)	21 (28.4)		24 (24.2)	30 (34.9)	
rs16942690	GG	9 (9.0)	13 (8.3)	0.981	7 (8.4)	10 (12.8)	0.367	7 (7.5)	13 (15.6)	0.296
	AG	38 (38.0)	70 (44.6)	0.388	42 (50.6)	32 (41.0)	0.300	46 (48.9)	37 (44.6)	0.994
	AA	53 (53.0)	74 (47.1)		34 (41.0)	36 (46.2)		41 (43.6)	33 (49.8)	
rs293381	TT	11 (11.0)	24 (15.0)	0.716	9 (10.5)	11 (14.5)	0.353	8 (8.1)	12 (14.0)	0.690
	CT	61 (61.0)	86 (53.8)	0.369	46 (53.5)	41 (54.0)	0.531	62 (62.6)	46 (53.5)	0.061
	CC	28 (28.0)	50 (31.2)		31 (36.0)	24 (31.5)		29 (29.3)	28 (32.5)	
rs12442260	CC	10 (10.0)	11 (7.0)	0.226	15 (17.4)	14 (17.5)	0.312	10 (10.2)	6 (7.0)	0.173
	CT	48 (48.0)	65 (41.4)	0.280	43 (50.0)	26 (32.5)	0.012	49 (50.0)	29 (33.7)	0.058
	TT	42 (42.0)	82 (51.6)		28 (32.6)	40 (50.0)		39 (39.8)	51 (59.3)	
rs729707	AA	27 (27.0)	43 (26.9)	0.176	23 (28.8)	23 (29.1)	0.686	29 (29.3)	27 (31.4)	0.244
	AG	61 (61.0)	86 (53.7)	0.765	49 (61.2)	46 (58.2)	0.862	59 (59.6)	43 (50.0)	0.872
	GG	12 (12.0)	31 (19.4)		8 (10.0)	10 (12.7)		11 (11.1)	16 (18.6)	

SNP, single-nucleotide polymorphism.

^a^ *P*-values adjusted by logistic regression for age, gender in non-smokers.

^b^ *P*-values adjusted by logistic regression for age, gender and pack-years in former and current smokers.

Association between haplotypes and diseases can, in some instance, highlight genetic influences that are not detected by SNP analysis. Linkage disequilibrium (LD) values among the 6 SNPs investigated were low, indicating that none of these SNPs was associated with each other (*R* ^2^ < 0.005). Thus, haplotypes could not be tested in the current study.

**Fig 1:**
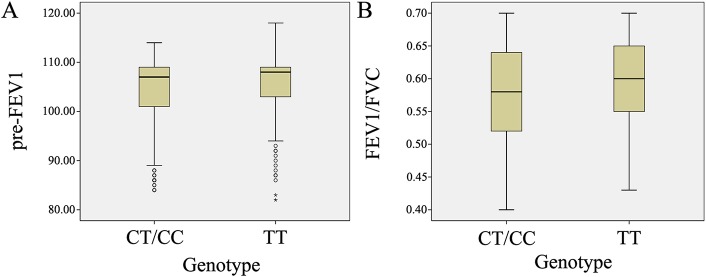
The association between *ABHD2* polymorphism rs12442260 and pulmonary function under a dominant model. (A) Rs12442260 is associated with pre-FVE1 in controls; (B) Rs12442260 is associated with FEV1/FVC ratio in patients with COPD.

To assess whether *ABHD2* polymorphisms were associated with pulmonary function in the Chinese Han population, a dominant model of genetic association analysis was performed for pre-FEV1 values and FEV1/FVC ratios using general linear models. Individual with the rs12442260 CT/CC genotype had a lower annual average in pre-FEV1 values than did those with the TT genotypes in 323 control subjects (TT: n = 172, 105.44±6.41, CT+CC: n = 151, 103.72±7.59; *P* = 0.027; Fig 1A), but this decline was not observed among 284 patients with COPD (TT: n = 109, 70.67±8.82, CT+CC: n = 175, 68.54±9.28; *P* = 0.071). When the analysis was conducted with the COPD group only, the results showed that the rs12442260 locus was associated with FEV1/FVC ratios in the COPD group (*P* = 0.012; Fig 1B), but not in the control group (*P* = 0.344). No significant association was evident between the other SNPs and indexes of pulmonary function (pre-FEV1 values and FEV1/FVC ratio).

## Discussion

Although cigarette smoking is considered to be an underlying risk factor for developing COPD, only 10 ~15% of chronic, heavy cigarette smokers develop symptomatic airflow obstruction [23]. As a complex disease with genetic-environmental interactions, COPD is one of the primary causes of morbidity and has become the fourth most common single cause of death [24–26]. Findings from GWAS studies, familial studies, and case-control studies, suggest that COPD susceptibility may be influenced by genetic factors [27, 28]. In the current study, we investigated the prevalence of *ABHD2* gene variants in 286 Chinese Han patients with COPD and 326 normal controls. Six SNPs, including rs293379, rs293377, rs16942690, rs293381, rs12442260, and rs729707, with minor allele frequencies greater than 10% and pairwise *R* ^*2*^ > 0.8 values were chosen.

To the best of our knowledge, this is the first study to implicate *ABHD2* SNPs risk factors for developing COPD. Results from previous studies have clearly demonstrated that *ABHD2* specifically expressed in alveolar type II cells [18] and SMCs [29]. *Abhd2*-deficient mice generated via gene trap insertional mutagenesis developed spontaneous, gradual emphysema. In addition, *Abhd2* deletions caused increases in SMC migration and intimal hyperplasia as well as changes in physiological functions caused by cuff placement, which are characteristic symptoms of COPD development in mouse models.

Our results revealed that *ABHD2* SNP at the rs12442260 locus that influence COPD risk in the Chinese Han population. Carriers of the C allele had an 83% increased risk of developing COPD compared with homozygotes, under a dominant genetic model (CC + CT vs. TT: OR = 1.83, 95% CI = 1.32–2.53; *P* < 0.001). In former smokers (n = 167), the rs12442260 polymorphism was significantly associated with COPD under the assumption of a dominant model of inheritance (CC + CT vs. TT: OR = 2.07, 95% CI = 1.10–3.89; *P* = 0.022). These results indicate that polymorphisms in *ABHD2* may contribute to the development of COPD in the Chinese Han population, and this association may be mediated by smoking behavior, although our data failed to identify this association in current smokers. However, differences in average smoking exposure (i.e. pack-years per subject) may be an important factor to explain this conflict. Rs293377 was also associated with COPD after adjusting for age, gender, and current smoking status (GC vs. CC: *P*_adjust_ = 0.018), but no correlation was found when comparing other genotypes (G-allele vs. C or GG vs. CC). A limitation of the study was that only one SNP was identified that was associated with COPD risk. In addition, this study did not provide evidence for associations between *ABHD2* haplotypes and the risk of COPD.

In a previous study, we provided evidence that *Abhd2* plays a critical role in maintaining lung structural integrity, providing a rationale for assessing whether *ABHD2* polymorphisms influence pulmonary function in the Chinese Han population in this study. Using a dominant model, only one of the SNPs studied was associated with pulmonary function. The Rs12442260 locus was associated with pre-FEV1 values in control subjects, but not in patients with COPD. This result also suggested that the risk of individuals in the control group with Rs12442260 polymorphisms for developing COPD may be affected by environmental factors. The Rs12442260 locus was also associated with FEV1/FVC ratios, but only in patients with COPD. No significant association was found between the other SNPs and the indexes of pulmonary function (pre-FEV1 values and FEV1/FVC ratios, data not shown). Another limitation of this study is that it is unclear whether rs12442260T variant genotypes affect the normal cellular function of ABHD2. Because the rs12442260 site is located within intron 5 of the ABHD2 gene, it potentially may affect ABHD2 mRNA splicing. An intron-located SNP (rs588076 SNP of the PICALM gene) has been shown to affect gene transcription and splicing [30], supporting the possibility that intronic rs12442260 SNPs may regulate ABHD2 mRNA splicing. Further studies will be needed to study the intronic regions containing regulatory elements, such as enhancer or attenuator elements, which potentially regulate ABHD2 gene transcription. Investigations into the abilities of regulatory genetic markers to predict pulmonary function risks in patients with COPD are essential.

In addition to the limitations mentioned above, this study had several strengths. This study is the first to show that ABHD2 polymorphisms are associated with COPD risk in a case-controlled manner. Using logistic regression analysis may delineate the influences of critical environmental and genetic factors. Furthermore, our sample size provided 70.0% power in detecting relative genetic risks. However, some limitations were inherent in this case-controlled study and should be noted. The sample size (286 patients with COPD and 326 control patients) was not comparatively large among most other COPD association studies published to date. This study was a hospital-based case-controlled study, meaning that there was the potential for information or selection bias. However, such bias was unlikely to be of significance because the controls were age- and sex-frequency matched with the COPD group. In addition, there is a potential concern that population admixture occurred, which could cause inflated type-I errors. To avoid this limitation, patients with COPD and control subjects were evaluated in the same hospital, and the ethnicity was limited to Han Chinese We selected target SNPs in HapMap with MAFs higher than 10% in CHB individuals to assess whether the ABHD2 gene was associated with COPD. In this study, smoker-status was the only environmental factor analyzed. Data from previous studies have suggested that COPD is a complex polygenic disease that is caused by the interaction of genetic and environmental factors. Further studies must consider other environment factors such as, for example, aerial pollution.

In conclusion, we first performed a comprehensive analysis of SNPs in the ABHD2 gene in a Chinese Han population. Our data revealed that the rs12442260 variant in the ABHD2 gene and its interaction with former smoking status were associated with COPD risk and may be important for pre-FEV1 and the FEV1/FVC ratios in the general population. The results of our study could help define the role of this polymorphism as a risk factor for developing of COPD.

## Methods

### Study population

A total of 286 patients with COPD and 326 normal control patients were enrolled between June 2011 and April 2013 at the Division of Respiratory Disease in the Fourth Hospital of Harbin Medical University of Harbin, China. Control patients were randomly selected from a health check-up program, had normal lung function, and were age- and gender-frequency matched with COPD cases. None of the subjects shared kinship with each other, nor were they related to the Han Chinese ethnic group from the Heilongjiang province in northeast China. As recommended in the Global Initiative for Chronic Obstructive Lung Disease Criteria Guidelines, patients with COPD were included in this study using the following criteria: age ≥ 40 years, a post-bronchodilator forced expiratory volume in 1 s (FEV1)/forced vital capacity (FVC) ratio < 0.70, an FEV1 of < 80% of the predicted value, and no other respiratory diseases [8]. All participants donated 5 ml peripheral blood after providing written, informed consent to participate in this study. This study was approved by the Ethics Committee of the Fourth Hospital of Harbin Medical University and was conducted according to the Declaration of Helsinki Principles.

### SNP selection and genotyping analysis

**Fig 2:**
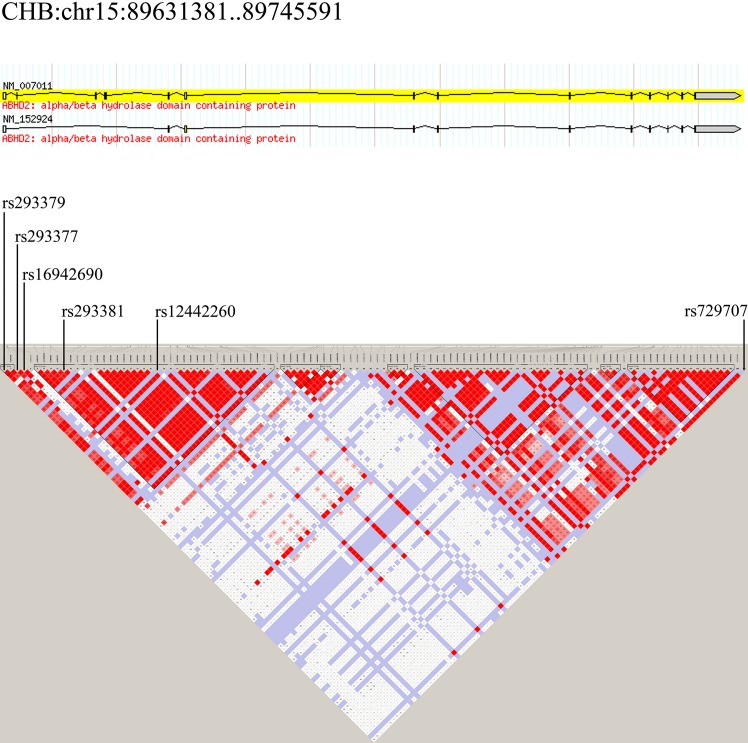
Haplotype block and LD structure for SNPs in Chr15: 89631381–89745591 in Chinese Han Beijing population.

Genomic DNA was extracted from whole blood samples using the Gentra Puregene DNA Extraction Kit (QIAGEN), according to the manufacturer’s instructions. SNPs were selected using Haploview Tagger [31] and based on CHB data using the following criteria: MAF > 10% and pairwise R2 > 0.8 (Fig 2). Linkage disequilibrium blocks were determined using data from HapMap data release #24 (November 2008; NCBI B36 assembly; dbSNP b126). Six SNPs (rs293379, rs293377, rs16942690, rs293381, rs12442260, and rs729707) located on 15q26.1 were chosen for the ABHD2 gene (S1 Table and S2 Table). SNP genotyping was performed by the sequence-specific priming (PCR-SSP) method. The primer sequences and amplified fragment lengths are listed in S3 Table. DNA samples were sequenced using a 3730 DNA Analyzer (Applied Biosystems Inc., USA), as shown in S1 Fig.

### Statistical analysis

Gene allele frequencies were measured in samples from patients with COPD and control patients. Continuous data were presented as the mean ± standard deviation (SD). Differences in demographic characteristics and selected variables observed between patients with COPD and controls were evaluated using the χ2 test (for categorical variables) and the Student’s t-test (for continuous variables). The Hardy-Weinberg equilibrium (HWE) for the control groups was assessed using the chi-square goodness of fit. Under the unconditional logistic regression statistic model with adjustments for age, gender, pack-years, and smoking status, the associations between genotypes and COPD risk were estimated by calculating genotype-specific odds ratios (ORs) and 95% confidence intervals (CIs). We also performed a smoking status-stratified analysis to eliminate the potential impact of cigarettes. In addition, linear regression was performed to assess relationships between SNPs and quantitative phenotypes (pulmonary function). Statistical manipulations were undertaken using the SPSS statistical analysis program, Version 19. P-values less than 0.05 were considered statistically significant for all analyses. To estimate linkage disequilibrium (LD) between pairs of loci in the patient and control populations, a standardized disequilibrium coefficient (D’) and squared correlation coefficient (R2) were calculated using Haploview 4.2 (www.broad.mit.edu/mpg/haploview/) [32]. LD blocks were defined in accordance with Gabriel’s criteria [33]. The statistical powers of the study were calculated under the assumption of a small effect size (0.1) with an alpha level of significance of 0.05 [34].

## Supporting Information

S1 Fig Genotyping of six SNPs by direct sequencing. (TIF)

S1 Table Raw genotype data of 286 patients with COPD. (XLSX)

S2 Table Raw genotype data of 326 control. (XLSX)

S3 Table Markers genotyped in the current study. (DOC)
