# Supplementary figures and images for: Associations of ABHD2 Genetic Variations with Risks for Chronic Obstructive Pulmonary Disease in a Chinese Han Population
Source: PLoS One. 2015 Apr 16;10(4):e0123929. doi: 10.1371/journal.pone.0123929 (PMC4399978; doi:10.1371/journal.pone.0123929)

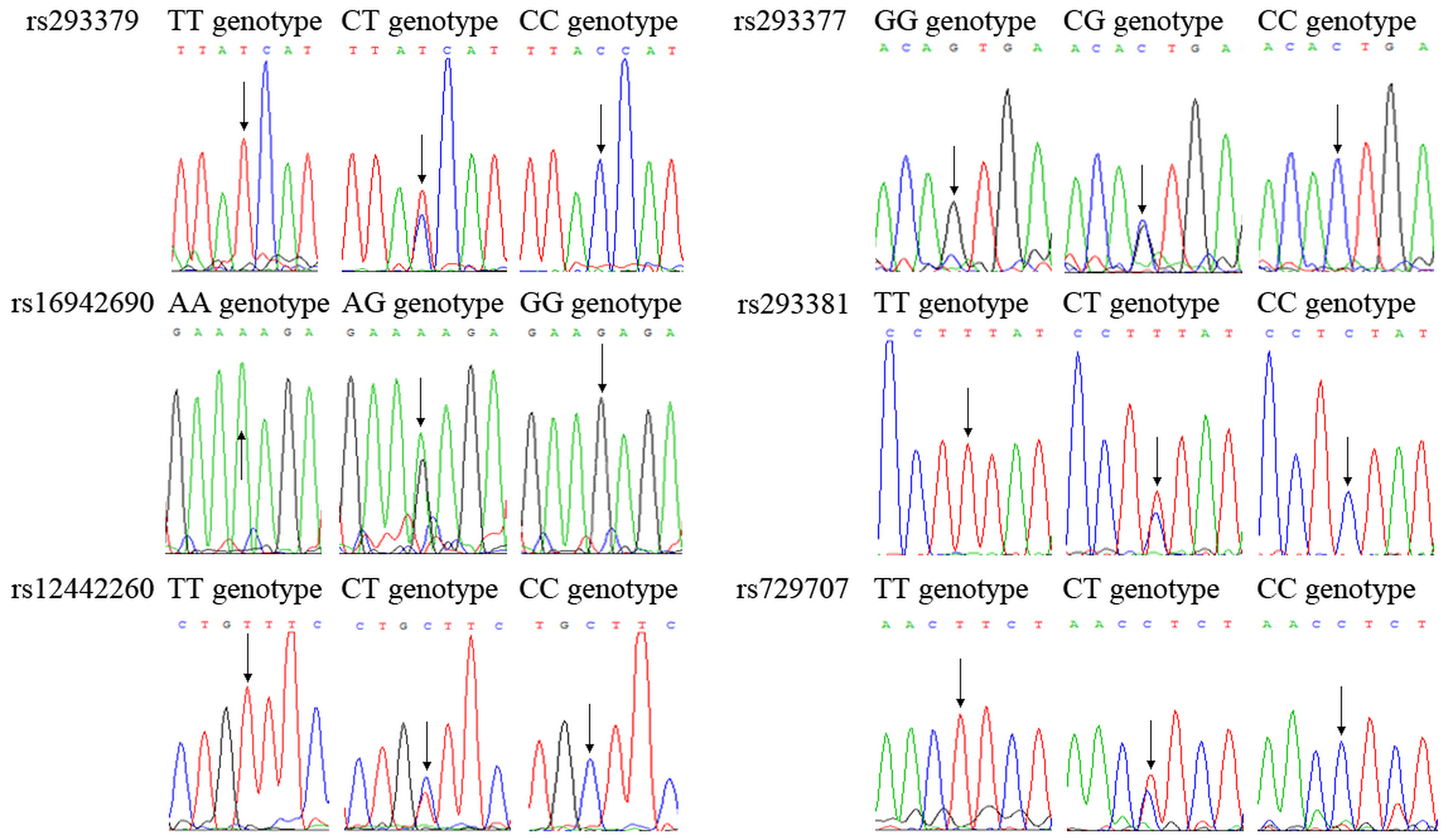

Supplement: S1 Fig — (TIF) [file pone.0123929.s001.tif]
