# Supplementary material for: Associations of ABHD2 Genetic Variations with Risks for Chronic Obstructive Pulmonary Disease in a Chinese Han Population
Source: PLoS One. 2015 Apr 16;10(4):e0123929. doi: 10.1371/journal.pone.0123929 (PMC4399978; doi:10.1371/journal.pone.0123929)
Supplement: S3 Table — (DOC) [file pone.0123929.s004.doc]

**Table S3. Markers genotyped in the current study.**

| NCBI rs number | Position | Gene redion | Genotype | F-PCR primer | R-PCR primer |
| --- | --- | --- | --- | --- | --- |
| rs293379 | 89633940 | Intron | C/T | TGCCTATTTGTCAGACCCAC | TCCCTTGTACTTGCCATC |
| rs293377 | 89634414 | Intron | G/C | TTCAGCCCTCCTCCCAAGC | ACCGAAATTCAGAATCAACTCA |
| rs16942690 | 89634442 | Intron | A/G | TTCAGCCCTCCTCCCAAGC | ACCGAAATTCAGAATCAACTCA |
| rs293381 | 89644938 | Intron | C/T | GCCCAATGTAATAATCTG | AAGCATTTACTTGGCTAC |
| rs12442260 | 89656467 | Intron | T/C | ATGGTGATTAAGAGGAGGAT | TCCAGAAATGCCTAACAG |
| rs729707 | 89743043 | Exon | A/G | TTCCTCAAGTGGCCTGTA | GAAAGCTCTACCCACATACA |

F-forward; R-reverse.
